# Wnt3a Promotes the Vasculogenic Mimicry Formation of Colon Cancer via Wnt/β-Catenin Signaling

**DOI:** 10.3390/ijms160818564

**Published:** 2015-08-10

**Authors:** Lisha Qi, Wangzhao Song, Zhiyong Liu, Xiulan Zhao, Wenfeng Cao, Baocun Sun

**Affiliations:** 1 Department of Pathology, Tianjin Medical University Cancer Institute and Hospital, Tianjin 300060, China; E-Mails: qilisha2005@163.com (L.Q.); baoer778@139.com (W.S.); liuzhiyong@tjmuch.com (Z.L.); 2 The Key Laboratory of Tianjin Cancer Prevention and Treatment, Tianjin 300060, China; 3 National Clinical Research Center for Cancer, Tianjin 300060, China; 4 Department of Pathology, Tianjin Medical University, Tianjin 300070, China; E-Mail: zhaoxiulan2014@163.com

**Keywords:** Wnt/β-catenin signaling, vasculogenic mimicry, Wnt3a, Dkk1, colon cancer

## Abstract

Our previous study provided evidence that non-canonical Wnt signaling is involved in regulating vasculogenic mimicry (VM) formation. However, the functions of canonical Wnt signaling in VM formation have not yet been explored. In this study, we found the presence of VM was related to colon cancer histological differentiation (*p* < 0.001), the clinical stage (*p* < 0.001), and presence of metastasis and recurrence (*p* < 0.001). VM-positive colon cancer samples showed increased Wnt3a expression (*p* < 0.001) and β-catenin nuclear expression (*p* < 0.001) compared with the VM-negative samples. *In vitro*, over-regulated Wnt3a expression in HT29 colon cancer cells promoted the capacity to form tube-like structures in the three-dimensional (3-D) culture together with increased expression of endothelial phenotype-associated proteins such as VEGFR2 and VE-cadherin. The mouse xenograft model showed that Wnt3a-overexpressing cells grew into larger tumor masses and formed more VM than the control cells. In addition, the Wnt/β-catenin signaling antagonist Dickkopf-1(Dkk1) can reverse the capacity to form tube-like structures and can decrease the expressions of VEGFR2 and VE-cadherin in Wnt3a-overexpressing cells. Taken together, our results suggest that Wnt/β-catenin signaling is involved in VM formation in colon cancer and might contribute to the development of more accurate treatment modalities aimed at VM.

## 1. Introduction

Solid tumor formation is highly dependent on a continuous supply of oxygen and nutrition. Angiogenesis has a vital function in tumor development because tumors are supplied with oxygen and nutrients and tumor cells are provided with an entry route into circulation. For years, much attention has been focused on the function of sprouting angiogenesis or the recruitment of endothelial cells into tumors from surrounding, pre-existing blood vessels. However, over the last few years, several other tumor microcirculation patterns have been identified, including vessel co-option, recruitment of endothelial precursor cells, intussusception, and vasculogenic mimicry (VM) [1,2,3]. VM was first described in 1999 by Maniotis *et al.* [4], who found that uveal melanoma cells resembled the endothelial phenotype and form vascular networks in the absence of endothelial cells. Although the functionality and contribution of VM have been established, many studies involving the use of intravenously injected tracers, laser-scanning confocal angiography, or doppler ultrasonography have indicated that VM can provide blood and nutrition to tumor masses even in the absence of conventional endothelial vessels [5,6,7]. In the last 10 years, VM has been observed in many different tumor types, and its occurrence is strongly associated with unfavorable clinical outcomes [8,9,10,11,12,13]. Furthermore, the conventional anti-angiogenesis treatment aimed at endothelial cells cannot inhibit VM formation. Several recent studies have indicated that anti-angiogenesis modalities may even elicit a more aggressive tumor phenotype [14,15]. Therefore, such conventional treatment strategies need to be reconsidered, and studies on the molecular mechanisms underlying VM are needed to develop more effective treatment modalities.

Wnt signaling is involved in a wide range of physiological processes, including embryonic development, cell proliferation, and homeostasis. Activating the mutations of the Wnt-signaling pathway is crucial in tumorigenesis, especially in colorectal cancer. Wnt signaling also has an important function in vascular development and angiogenesis. Previous reports have shown that Wnt signaling contributes to endothelial cell differentiation and vascular remodeling [16]. Both the loss and gain of function of Wnt pathway may influence endothelial cell functions and result in abnormal vascular development and angiogenesis [17,18]. Signaling through the Wnt pathway starts with Wnt ligands, which consist of more than 19 cysteine-rich glycoproteins. Constitutive activation of Wnt signalling through mutation of APC, β-catenin or Axin appears to be a necessary initiating step for more than 85% colorectal cancers. However, emerging evidence demonstrates that the upstream components (*i.e.*, some Wnt ligands and Frizzled) are overexpressed in colon cancer and are involved in tumor progression [19,20]. Additionally, some recent studies demonstrate that inhibitors of Wnt-Frizzled interaction could suppress tumor growth and development [21,22]. All these indicate the additional modulation of Wnt signalling via the upstream components, despite mutations in the downstream components of the pathway. Particular Wnt ligands, such as Wnt1, Wnt3a, and Wnt7a, stimulate the β-catenin dependent pathway; this stimulation is called Wnt/β-catenin or canonical Wnt signaling. Other ligands, such as Wnt4, Wnt5a, and Wnt11, may activate the Ca^2+^-calmodulin kinase or the planar cell polarity pathway; this activation is called non-canonical Wnt signaling. Our previous reports have shown that overexpression of Wnt5a, a representative non-canonical Wnt ligand, may mediate VM formation in ovarian cancer and non-small cell lung cancer [23,24]. By contrast, data on canonical Wnt signaling in VM formation are still scarce.

Wnt3a is a representative Wnt protein that signals via Wnt/β-catenin signaling. Previous studies, including our own, have reported that by activating Wnt/β-catenin signaling, Wnt3a can promote epithelial-mesenchymal transition and enhance stemness of cancer cells [20], both of which are reported as important mechanisms underlying VM. Thus, we speculate that Wnt3a may be involved in regulating VM formation. In this study, the expression patterns of Wnt3a, β-catenin, and VM were examined on a large array of 217 human colon cancer cases. The relationships between Wnt3a, β-catenin, and VM were explored. The effects of ectopic expression of Wnt3a in colon cancer cell line HT29 on the tube-structure forming ability and VM-associated proteins *in vitro* as well as on the VM forming ability in animal xenograft model were studied. In addition, we treated Wnt3a-overexpressing cells with Dkk1, a Wnt/β-catenin pathway antagonist, and determined the tube-structure forming ability and expression of VM-related proteins to further verify the VM- promoting effect of the Wnt/β-catenin signaling pathway.

## 2. Results

### 2.1. Association of VM Frequency with Clinicopathological Features of Colon Cancer Cases

**Figure 1:**
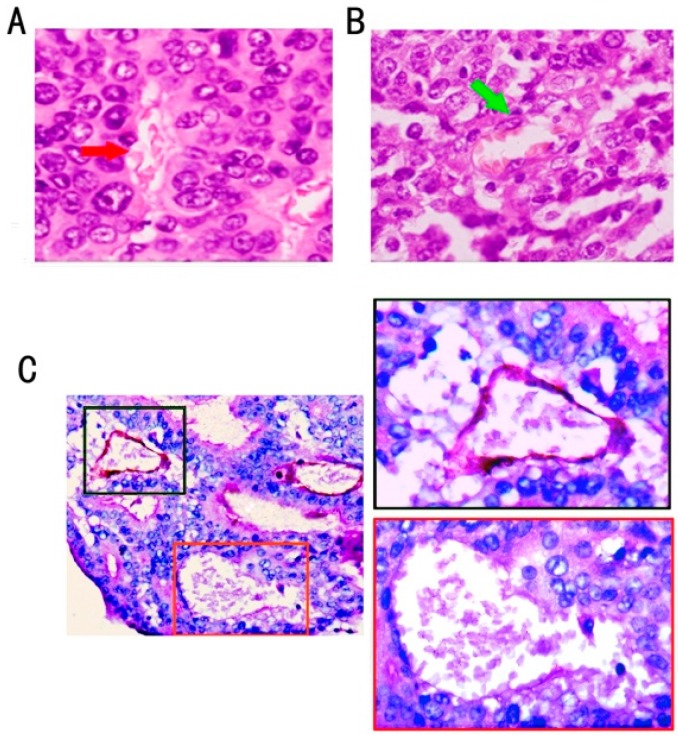
VM structure and endothelial-dependent vessels in colon cancer. (**A**) VM channel (red arrow) lined with tumor cells and containing red blood cells (H&E staining, ×400); (**B**) Endothelial-dependent vessel lined with flat endothelium cells (green arrow) (H&E staining, ×400); (**C**) VM channel formed by tumor cells was negative for CD34; the one lined with a base membrane-like structure was positive for PAS (red square frame); and the endothelial-dependent vessel was both positive for CD34 and PAS (black square frame) (CD34/PAS double staining, ×200).

Using H&E staining (Figure 1A,B) and CD34/PAS double-staining (Figure 1C), VM was distinguished by channels lined with colon cancer cells instead of shuttle-like endothelial cells. The VM channel showed a positive expression for PAS but a negative expression for CD34, confirming that cells around the channels were not composed of endothelium. Red blood cells were found inside the VM channels. No necrotic and infiltrating inflammatory cells were observed around the channels. The endothelial-dependent vessels were positive for CD34.

VM was detected in 39 (19.2%) out of 217 colon cancer cases. The clinical and pathological features of VM in all 217 colon cancer cases are summarized in Table 1. The presence of VM was strongly correlated with histological differentiation (*p* < 0.001), TNM stages (*p* < 0.001), and metastasis/recurrence (*p* < 0.001). The frequency of VM was significantly higher in poorly differentiated colon cancer (30/53, 56.6%) than in well (1/14, 7.1%) and moderately (8/109, 7.3%) differentiated ones. VM was observed in 33 of 69 patients (47.8%) with advanced stage carcinomas (TNM stages III and IV), in 16 of 148 patients (10.8%) with early-stage carcinomas (TNM stages I and II). A total of 77 (35.5%) colon cancer patients experienced metastasis or recurrence. The patients with VM had a higher rate of metastasis or recurrence (24/77, 31.2%) than those ones without VM (15/77, 19.5%). No significant correlations were found between VM and patient age or gender, tumor location or size.

**Table 1:** Correlation between VM and clinicopathologic characteristics of colon cancer and expression of Wnt3a and β-catenin.

Variable	Total (%)	Tissue Samples				χ^2^	*p* Value	
		VM (%)		nonVM (%)				
Age								
<45	28 (12.9)	7 (25.0)		21 (75.0)		1.077	0.298	
≥45	189 (87.1)	32 (16.9)		157 (83.1)				
Sex								
Male	101 (46.5)	19 (18.8)		82 (81.2)		0.09	0.86	
Female	116 (53.5)	20 (17.2)		96 (82.8)				
Location								
Left hemicolon	125 (57.6)	25 (20.0)		100 (80.0)		0.475	0.822	
Right hemicolon	92 (42.4)	14 (15.2)		78 (84.8)				
Tumor size(cm)								
≥10	25 (11.5)	6 (24)		19 (76)		0.70	0.278	
<10	192 (88.5)	33 (17.2)		159 (82.8)				
Histological differentiation								
Well differentiated	14 (6.4)	1 (7.1)		13 (92.9)		72.11	<0.001 *	
Moderately differentiated	109 (50.2)	8 (7.3)		101 (92.7)				
Poorly differentiated	53 (24.4)	30 (56.6)		23 (43.4)				
Mucinous carcinoma	41 (18.9)	0 (0.0)		41 (100.0)				
TNM stage									
TNMⅠ	10 (4.6)	0 (0.0)		10 (100.0)		23.20	<0.001 *	
TNMⅡ	138 (63.6)	16 (11.6)		122 (88.4)				
TNMⅢ	57 (26.3)	16 (28.1)		41 (71.9)				
TNMⅣ	12 (5.5)	7 (58.3)		5 (41.7)				
Metastasis/recurrence									
Present	77 (35.5)	24 (31.2)		53 (68.8)		14.099	<0.001 *	
Abscent	140 (64.5)	15 (10.7)		125 (89.3)				
Wnt3a expression									
Negative	22(10.1)	0(0.0)		22(12.4)		17.7	<0.001 *	
Weak expression	97(44.7)	10(34.5)		87(48.9)				
Strong expression	98(45.2)	29(65.5)		69(38.7)				
β-catenin expression									
Nuclear negative	175 (80.6)	18 (46.2)		157 (88.2)		36.2	<0.001 *	
Nuclear positive	42 (19.4)	21 (53.8)		21 (11.8)				

* Significantly different.

### 2.2. VM Was Associated with Wnt3a Expression and reCatenin Nuclear Expression

**Figure 2:**
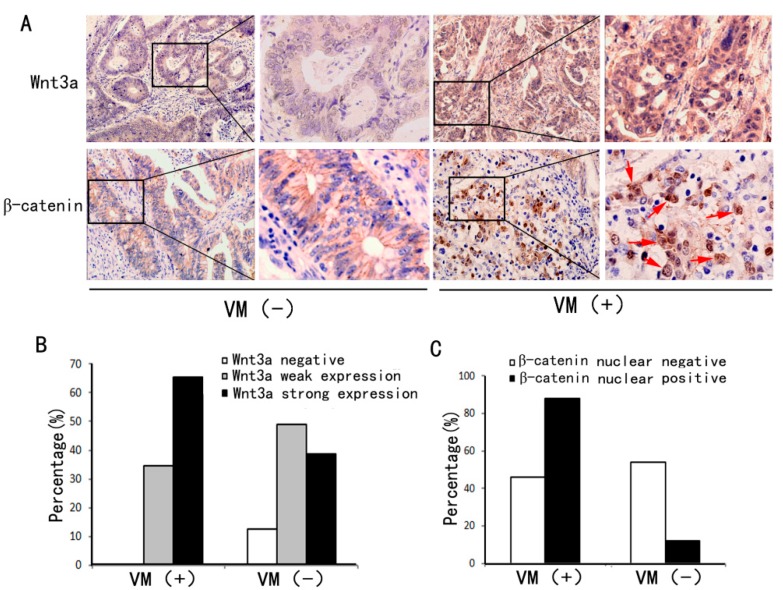
Expressions of Wnt3a and β-catenin in the VM-positive and VM-negative groups. (**A**) Wnt3a expression was higher in VM-positive colon cancer tissue sections (**right**) than in VM-negative samples (**left**). In VM-positive sections, the tumor cells displayed nuclear β-catenin accumulation (red arrows), whereas those in the VM-negative section showed only membranous localization of β-catenin (immunohistochemical staining, ×200); (**B**) Percentages of Wnt3a negative, weak, and strong expression in the VM-positive and VM-negative groups; (**C**) Percentages of β-catenin nuclear positive and negative expression in the VM-positive and VM-negative groups.

To assess the relationship between VM and Wnt/β-catenin signaling in colon cancer, we determined the expression of Wnt/β-catenin signaling-associated markers, including Wnt3a and β-catenin. The former is a representative canonical Wnt signaling ligand, and the latter is the key regulator of canonical Wnt signaling. As shown in Figure 2, the VM group showed higher Wnt3a expression and lower nuclear β-catenin expression than the non-VM group. In addition, in the non-VM group, β-catenin was expressed mainly in cytoplasm and membrane, whereas it was distributed in the nucleus in the VM group. Statistical analysis results (Table 1) revealed that the strong expression of Wnt3a was more frequently detected in the VM group than in the non-VM group (65.5% *vs.* 38.7%, *p* < 0.05). Meanwhile, the nuclear expression of β-catenin was more frequently found in the VM group than in the non-VM group (53.8% *vs.* 11.8%, *p* < 0.05). The results indicate that VM is associated with Wnt/β-catenin signaling activation in colon cancer.

### 2.3. Wnt3a Overexpression Induced the Activation of Canonical Wnt Signaling in HT29 Cells

**Figure 3:**
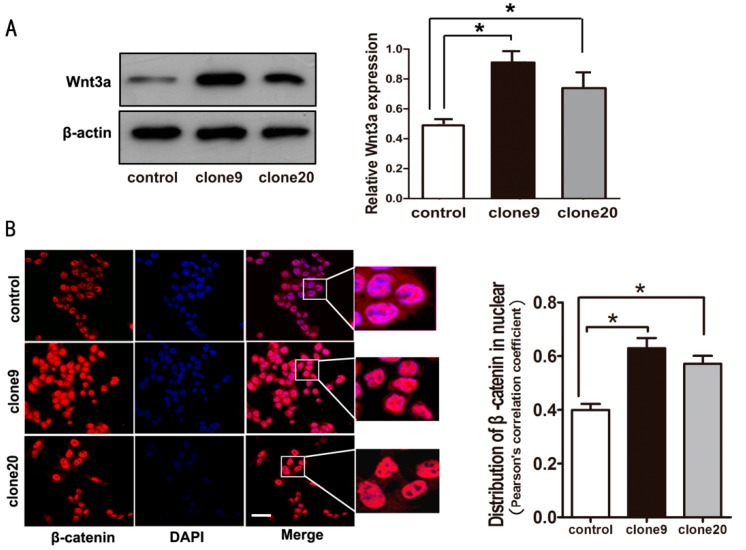
Wnt3a overexpression-induced activation of Wnt/β-catenin signaling in HT29 cells. (**A**) Wnt3a protein expression levels were significantly increased in clone9 and clone20, and HT29 cell pools transfected with Wnt3a plasmid (**left**). Relative amount of protein expression of Wnt3a/β-actin (**right**) (* *p* < 0.05); (**B**) Increased β-catenin accumulation in the nucleus was observed in cells overexpressing Wnt3a compared to control cells (immunofluorescent staining) (**left**). Scale bar: 50 μm. Red signal represents staining for β-catenin, and blue signal represents nuclear DNA staining by DAPI. Microscopic analysis of β-catenin with DAPI showed increased nuclear β-catenin distribution in cells overexpressing Wnt3a (**right**) (* *p* < 0.05).

Our previous study showed that HT29 cells, a better differentiated and less invasive cell line, cannot form vascular networks *in vitro* at 3-D culture conditions [25]. Thus, we used HT29 cells to investigate the effect of canonical Wnt signaling activation and VM formation. We established stable Wnt3a-overexpressed HT29 cells to further study the VM-promoting effect of canonical Wnt signaling on colorectal cancer cells. To rule out clone-to-clone variations, we selected two clones (clone9 and clone20, Figure 3A). Immunofluorescence results showed that more β-catenin was accumulated in the nucleus of cells overexpressing Wnt3a than in control cells (Figure 3B), thereby suggesting that HT29 cells overexpressing Wnt3a activated canonical Wnt signaling.

### 2.4. Wnt3a Overexpression Promoted the Tube-Like Structure Formation of HT29 Cells in Vitro and Promoted in Vivo Tumor Growth and VM Formation in Animal Models

**Figure 4:**
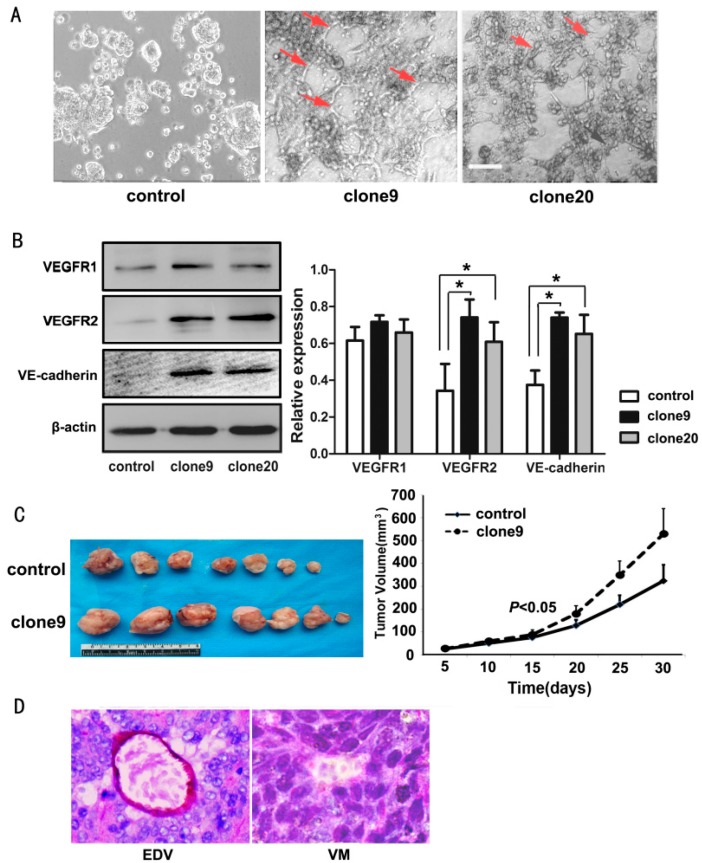
Wnt3a promoted the VM-forming ability in HT29 cells. (**A**) HT29 cells cannot form typical tube-like structures in the 3D culture, whereas Wnt3a-overexpressing clone9 and clone20 cells formed few tubular structures (red arrows). Scale bar: 100 μm; (**B**) Upregulated Wnt3a expression in HT29 cells results in increased VEGFR2 and VE-cadherin expressions. No significant change in VEGFR1 expression was observed (**left**) (* *p* < 0.05). Relative amount of protein expression of VEGFR1, VEGFR2, and VE-cadherin compared with β-actin (**right**); (**C**) Representative xenograft tumors of the control or Wnt3a-overexpressing HT29 cells (clone9) on the 30th day post injection (**left**). Tumor volumes are monitored over time (**right**); (**D**) VM structure and endothelial-dependent vessels in xenograft tumors (CD34/PAS double staining, ×400).

Three-dimensional culture is used to effectively test not only the vascular behavior of endothelial cells but also the ability of a number of tumor cells to form VM structures. We showed that HT29 cells cannot form tubular structures, whereas Wnt3a-overexpression HT29 cells (clone9 and clone20) formed few tubular structures, thereby indicating that Wnt3a may be an activator for VM formation in HT29 cells (Figure 4A).

Moreover, compared with the controls, the Wnt3a-overexpression cells showed increased expression of VE-cadherin and VEGFR2, which are the most representative VM-associated endothelial phenotype proteins. However, we did not observe a significant change in the VEGFR1 expression in the same cells (Figure 4B).

Wnt3a overexpressing cells grew into larger tumor masses compared with the control cells (*p* < 0.05) (Figure 4C). The VM counts of the xenograft mouse models were determined via CD34/PAS double staining. Similar to the *in vitro* findings, 3 of 10 tumor tissues from Wnt3a-overexpression HT29 cells (clone9) exhibited a VM structure, whereas all those from control cells showed no VM structures (Figure 4D), thereby further indicating the VM-promoting effect of Wnt3a on colorectal tumors.

### 2.5. Dkk1 Inhibited the Tube-Structure Formation in Vitro and Restored the VEGFR2 and VE-Cadherin Expression in Wnt3a-Upregulation HT29 Cells

**Figure 5:**
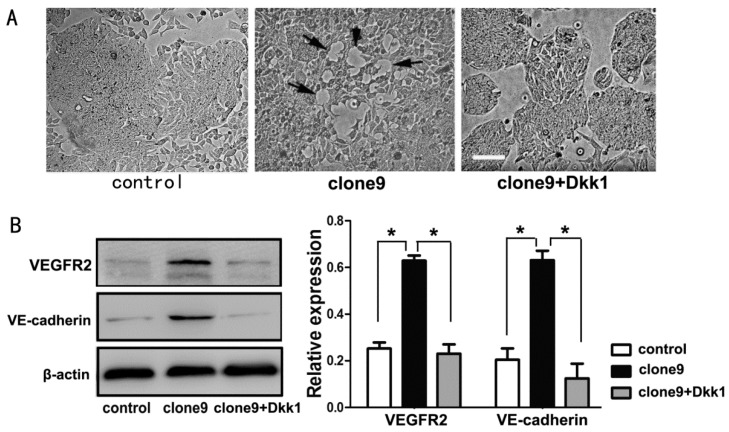
Dkk1 reversed the VM-forming ability in Wnt3a-overexpressing HT29 cells. (**A**) Dkk1 inhibited the *in vitro* tube-like structure formation (black arrows) of Wnt3a-overexpressing HT29 cells (clone9). Scale bar: 100 μm; (**B**) Dkk1 restored the expressions of VEGFR2 and VE-cadherin in clone9 cells (**left**). Relative amount of protein expressions of VEGFR2 and VE-cadherin compared with β-actin (**right**) (* *p* < 0.05).

Dkk1 functions as an antagonist of the Wnt/β-catenin pathway by binding to lipoprotein receptor-related protein 5 or 6 (LRP5/6) and preventing the formation of Wnt-Fz-LRP ternary complexes and downstream signaling transduction. After Dkk1 treatment, Wnt3a-overexpressing cells failed to form vessel-like tube formation (Figure 5A). Furthermore, Dkk1 treatment decreased the VEGFR2 and VE-cadherin expressions in Wnt3a-upregulation cells (Figure 5B). These findings further verified that Wnt/β-catenin pathway activation had an important function in the VM-inducing effect of Wnt3a.

## 3. Discussion

VM was first described in melanoma in 1999 [4], and this special tumor microcirculation pattern was observed in many different tumor types, including breast cancer, lung cancer, prostate cancer, ovarian cancer, synovial sarcoma, gastrointestinal stromal tumor, and glioblastoma [8,9,10,11,12,13]. VM describes the formation of a fluid-conducting vasculogenic-like network by tumor cells, particularly poorly differentiated ones. VM structures are composed of basement membranes that are stained positive with PAS and lined by tumor cells; no endothelial cells can be found on their inner wall. On one hand, VM may function as a complementary means to provide oxygen and nutrition to tumor masses independent of conventional endothelial vessels. On the other hand, the irregularly arranged tumor cells lining the lumen are directly exposed to the bloodstream. Thus, these cells can easily enter the microcirculation, which leads to distant dissemination. Except for its potential function in facilitating tumor cell metastasis to the blood stream and, subsequently, to distant organs, the tubular networks formed by highly aggressive tumor cells can contribute to tumor circulation because they appear to be capable of blood flow. Thus, the presence of VM in many tumors is strongly associated with poor clinical outcomes. In the present study, the presence of VM in colon cancer was characterized by histological differentiation and TNM stages. This result is consistent with the results of our previous study on colorectal carcinoma [25], in which we also indicated that VM is a prognostic marker for shorter survival.

A growing body of evidence has revealed the importance of Wnt/β-catenin signaling pathways in endothelial cell differentiation and vascular remodeling. During embryonic development, gain-of-function mutation of β-catenin in endothelial leads to vascular remodeling [26]. The activation of β-catenin can induce embryonic stem cells to differentiate into arterial endothelial cell [27]. Gherghe reported that the overexpression of Wnt1 in endothelial cells can elicit cellular responses that are critical for angiogenesis [28]. Blankesteijn observed that Wnt/β-catenin signaling is activated during pathological neovascularization but not in healthy, resting vasculatures [29]. Wnt/β-catenin signaling regulates the expression of angiogenic factors, including VEGF and IL-8 [30]. Although previous observations have shown the capacity of canonical Wnt signaling in regulating endothelial-dependent vessel formation, the present study provides the first clinical and experimental evidence that confirms the intimate link between Wnt/β-catenin signaling and VM formation. In human colon cancer tissue samples, higher levels of Wnt3a expression and β-catenin nuclear distribution were observed in the VM positive group than in the negative group. We further isolated stable Wnt3a-overexpressing HT29 cell clones and found that Wnt3a increased the intracellular distribution of β-catenin, which is an indicator of activated canonical Wnt signaling. More importantly, the ectopic expression of Wnt3a resulted in increased capacity to form tube-like structures on Matrigel *in vitro* and increased VM incidence in mouse-transplanted tumors. All these results indicated the VM-inducing effect of Wnt/β-catenin pathway in colon cancer.

VM repeats the pattern of embryonic vasculogenic networks. Molecular profiling of cells capable of VM showed that a number of highly upregulated genes are involved in angiogenesis and vasculogenesis [31]. When VEGF-A binds in an autocrine or paracrine manner, VEGFR2 primarily regulates endothelial cell differentiation, survival, proliferation, and migration. Moreover, the expression of VEGFR2 has been described as associated with VM formation [32]. VE-cadherin, a member of the cadherin family specifically expressed in endothelial cells, has a pivotal function in vascular integrity and regulates endothelial cell assembly into tubular structures. VE-cadherin was also identified as an important factor in VM formation because tumor cells lacking VE-cadherin are incapable of forming VM [33]. In this study, the activation of Wnt/β-catenin signaling by ectopic Wnt3a expression promoted VM formation and also enhanced the expressions of VEGFR2 and VE-cadherin, thereby suggesting that Wnt/β-catenin signaling may help colon cancer cells acquire a differentiation potential for endothelial cells.

Wnt3a not only activates Wnt/β-catenin signaling but also functions simultaneously with signaling pathways such as FAK or TGFβ [34,35]. Dkk1 is a potent antagonist of the Wnt/β-catenin signaling pathway. Dkk1 functions by binding to LRP5/6, thereby interfering the formation of frizzled-LRP6 complexes and inhibiting TCF/LEF transcription. Our results showed that Dkk1 treatment significantly inhibited the formation of tube-like structures of Wnt3a-overexpressing HT29 cells *in vitro* and induced decreased expressions of VEGFR2 and VE-cadherin, thereby indicating that the VM-inducing effect caused by Wnt3a was due to the stimulative effect of Wnt3a on the Wnt/β-catenin pathway.

In conclusion, this study is the first to report on a previously unrecognized function of Wnt/β-catenin signaling as an important regulatory pathway for VM formation. The results might have a number of implications for clinically useful therapy targets aimed at VM in the future.

## 4. Materials and Methods

### 4.1. Tissue Samples

In the current study, 217 formalin-fixed, paraffin-embedded human colon cancer tissue samples were from Tianjin Medical University Cancer Institute and Hospital from July 2002 to June 2004. All the patients did not receive chemotherapy or radiotherapy before operation. Data of clinical parameters were obtained from patients’ clinical records. The pathological diagnosis of all cases were independently verified by two senior pathologists by observing the H&E sections conserved in Pathology Department of Tianjin Medical University Cancer Institute and Hospital.

### 4.2. Cells and Reagents

Human colon cancer cell line HT29 was obtained from Cell Resource Center, Institute of Basic Medical Sciences, Chinese Academy of Medical Sciences, Peking Union Medical College (Beijing, China). HT29 cells were cultured in DMEM/F12 medium supplemented with 5% fetal calf serum (FCS) at 37 °C and 5% CO_2_. Antibodies for Dkk1, CD34, VEGFR1, VEGFR2 and β-actin were from Santa Cruz (CA, USA). Antibodies for Wnt3a, VE-cadherin and β-catenin were from Abcam (Cambridge, UK). The secondary HRP-conjugated antibodies were from Zhongshan Chemical Co. (Beijing, China). Goat anti-mouse IgG-FITC were from Santa Cruz. The eukaryotic expression vector GV230 containing whole coding sequence of Wnt3a was original obtained from Genechem Technology (Shanghai, China) (The plasmid contains a GFP tag and is neomycin resistant). In this study, the coding sequence of Wnt3a was amplified via PCR using the following primers: forward primer 5ʹ-GAG GAA TTC ATG GCC CCA CTC GGA TAC-3ʹ which contains an EcoR I recognition site, reverse primer 5ʹ-GAC AGC TCG AGCTA CT TGC AGG TGT GCA CGT CGT AG-3ʹ, which contains an Xho I recognition site, and then subcloned into vector pcDNA3.1 (+) eukaryotic expression vector. The recombinant plasmid was verified by DNA sequencing and double enzyme digestion (EcoR I and Xho I). Matrigel was from BD Biosciences (San Jose, CA, USA). BALB/C nude mice (4 to 6 weeks old) were obtained from Wei Tong Li Hua Experimental Animal Co., Ltd. (Beijing, China). Dkk1 recombinant protein was from R&D Systems (South Logan, UT, USA). For Dkk1 administration *in vitro*, Dkk1 (1 μg/mL) was added to culture medium. Fifty percent of the medium was replaced with fresh conditioned medium containing Dkk1 every 24 h. After 48 h Dkk1 treatment, cells were harvested and the total cell lysates were collected for measurement by Western blot.

### 4.3. Tissue Immunohistochemical Analysis

The streptavidin-biotin-peroxidase immunohistochemical staining method was used in this study. Briefly, after antigen retrieval for 20 min in citrate buffer (0.01 M citric acid, pH 6.0) at room temperature, sections were incubated overnight at 4 °C with the primary specific antibody (Wnt3a 1:100, and β-catenin 1:50). Then secondary HRP-conjugated antibody was used for 1h at room temperature to immunostained the sections and 3,3ʹ-diaminobenzidine buffer was used as substrate to reveal the signals. The sections were scored blindly by two pathologist using a microscope at 200× magnification. Wnt3a immunohistochemical staining was evaluated by assessing staining intensity and density established by Qi *et al.* [20]. It was considered nuclear β-catenin-positive expression if more than 10% percentage of tumor cells showed brown granules in nuclei.

### 4.4. CD34/Periodic Acid Schiff Double Staining

After immunohistochemical staining for CD34 (1:100), the sections were washed with running distilled water for 5 min and incubated with periodic acid for 15 min and Schiff reagent. Finally, all of the sections were counterstained with hematoxylin, dehydrated with ascending grade ethanol and mounted.

### 4.5. Plasmid Transfection

Transfection with plasmid carrying Wnt3a and controlled scrambled plasmid was performed with Lipofectamine according to the manufacturer’s instructions. HT29 cells were seeded in a 6-well plate (3 × 10^5^ cells/well). After 16 h at approximately 60% confluence, the cells were transfected with Wnt3a plasmid (4 μg/well) in 10 μL of transfection reagent in a final volume of 2 mL of transfection medium. Four hours after transfection, full culture medium without antibiotics was added to the mixture. Twenty-four hours after transfection, neomycin-resistant cells were screened to establish stable HT29 cells that overexpressed Wnt3a.

### 4.6. Western Blot Analysis

Total protein (35 μg) was resolved by 10% SDS-PAGE and transferred by electroblotting onto PVDF membrane (Millipore, Temecula, CA, USA). Blots were blocked for 1 h and incubated with primary antibodies (Wnt3a 1:500, VEGFR1 1:200, VEGFR2 1:200, VE-cadherin 1:500 and β-actin 1:2000) overnight at 4 °C. Subsequently, blots were washed in TBS containing 0.1% Tween20 and labeled with goat anti-mouse or goat anti-rabbit IgG-HRP (1:5000; Santa Cruz Biotechnology). Immunoreactive bands were visualized on an autoradiography film (Blue X-Ray Film, Phoenix Research, Candler, NC, USA), and the relative density of bands was analyzed using an Odyssey infrared scanner (LI-COR Bioscience, Lincoln, NE, USA).

### 4.7. Immunofluorescence

Cells were plated on sterile glass cover slips 1 day prior to staining. Cells were fixed with 10% formalin in PBS for 10 min, quenched with 50 mM NH_4_Cl for 10 min and 0.2% triton for 10 min. Then, after blocking with 3% BSA for 1 h, the slips were incubated overnight with anti-β-catenin (1:50) at 4 °C. Slides were washed in PBS and labeled with secondary antibodie for 1 h in the dark at room temperature. After immunolabeling, slides were washed, counterstained with DAPI and viewed with fluorescent microscopy (Olympus, Tokyo, Japan). Colocalization efficiency of DAPI and β-catenin was calculated through Image J software (NIH, Bethesda, MD, USA). The nuclear regions dyed with DAPI were selected as the region of interest to quantify the nuclear distribution efficiency of β-catenin. Eighteen images were analyzed.

### 4.8. In Vitro Three-Dimensional (3-D) Coculture

VM-forming ability was tested by using 3-D culture *in vitro*. Matrigel (0.1 mL/well) was applied on the twenty-four-well culture plate and incubated at 37 °C for half an hour. The cells were trypsinized and suspended in the complete medium at 2.5 × 10^5^ cells/mL, plated onto the surface of Matrigel at 1 mL/well, and incubated at 37 °C for 48 h.

### 4.9. Xenograft Mouse Model

Twenty mice were randomly divided into two groups and received either 3 × 10^6^ control cells or clone9 cells by subcutaneous injection in the right groin. Tumor volumes were measured every 5 days. All mice were killed on the 30th day post injection. Tumor masses were excised, fixed in formalin, embedded in paraffin, and then were subjected to H&E staining and CD34/PAS double staining.

### 4.10. Statistical Analysis

SPSS 16.0 software (SPSS Inc., Chicago, IL, USA) was used for data statistical analysis. *p* < 0.05 were considered statistically significant. The associations between VM and clinicopathologic parameters and the differential expression of Wnt3a and β-catenin between groups were compared using Chi-squared or Fisher’s exact tests in human samples. Differences between groups were assessed by the Mann-Whitney U-test and Student’s *t*-test.

## 5. Conclusions

Wnt/β-catenin signaling is involved in VM formation in colon cancer and might contribute to the development of more accurate treatment modalities aimed at VM.
